# Conformational Masking and Receptor-Dependent Unmasking of Highly Conserved Env Epitopes Recognized by Non-Neutralizing Antibodies That Mediate Potent ADCC against HIV-1

**DOI:** 10.3390/v7092856

**Published:** 2015-09-18

**Authors:** George K. Lewis, Andrés Finzi, Anthony L. DeVico, Marzena Pazgier

**Affiliations:** 1 Division of Vaccine Research, Institute of Human Virology, University of Maryland School of Medicine, 725 West Lombard Street, Baltimore, MD 21201, USA; adevico@umaryland.edu (A.L.D.V.); mpazgier@umaryland.edu (M.P.); 2 Centre de Recherche du CHUM, Department of Microbiology, Infectiology, and Immunology, Université de Montréal, Montreal, QC H2X 0A9, Canada; andres.finzi@umontreal.ca

**Keywords:** Fc, antibody, HIV-1, protection, effector-function, AIDS, vaccine

## Abstract

The mechanism of antibody-mediated protection is a major focus of HIV-1 vaccine development and a significant issue in the control of viremia. Virus neutralization, Fc-mediated effector function, or both, are major mechanisms of antibody-mediated protection against HIV-1, although other mechanisms, such as virus aggregation, are known. The interplay between virus neutralization and Fc-mediated effector function in protection against HIV-1 is complex and only partially understood. Passive immunization studies using potent broadly neutralizing antibodies (bnAbs) show that both neutralization and Fc-mediated effector function provides the widest dynamic range of protection; however, a vaccine to elicit these responses remains elusive. By contrast, active immunization studies in both humans and non-human primates using HIV-1 vaccine candidates suggest that weakly neutralizing or non-neutralizing antibodies can protect by Fc-mediated effector function, albeit with a much lower dynamic range seen for passive immunization with bnAbs. HIV-1 has evolved mechanisms to evade each type of antibody-mediated protection that must be countered by a successful AIDS vaccine. Overcoming the hurdles required to elicit bnAbs has become a major focus of HIV-1 vaccine development. Here, we discuss a less studied problem, the structural basis of protection (and its evasion) by antibodies that protect only by potent Fc-mediated effector function.

## 1. Introduction

Antibody-mediated protection against HIV-1 [1,2], HIV-2/SIV [3] or SHIV [4,5,6,7,8,9,10,11,12,13,14,15] has been demonstrated repeatedly over the last twenty-five years by passive immunization of non-human primates (NHP) with neutralizing antibodies. The recent discovery that some HIV-1 infected people mount clonal broadly neutralizing (bnAb) antibody responses [16,17] has spurred the search for vaccine to elicit bnAbs to prevent HIV-1 transmission. Although a bnAb-based vaccine has become the Holy Grail of HIV-1 vaccine development, it faces significant obstacles in the unusual genetic and structural requirements for neutralization fitness (reviewed or discussed in [18,19,20,21]). Here, we discuss the structural basis of a complementary mechanism of antibody-mediated protection against HIV-1; potent Fc-mediated effector function by non-neutralizing antibodies. In contrast to neutralizing antibodies, passive immunization with non-neutralizing antibodies that can only work by Fc-mediated effector function have failed to protect against high-dose SHIV challenges in NHPs, although post-infection control of viremia was observed ([14,22,23], and in preparation). An early report suggested that non-neutralizing antibodies with potent Fc-mediated effector function could passively protect against SIV [24,25] but this was not seen a following study [26]. Taken at face value, these studies seem to disfavor a role of Fc-mediated effector function alone in antibody-mediated protection against HIV-1. However, a large body of literature, ranging from vaccine studies in NHPs and humans to natural infection cohorts, has consistently correlated Fc-mediated effector function by non-neutralizing antibodies with protection against HIV-1 (reviewed in [27,28,29,30,31]). In the following paragraphs, we discuss the structural basis of an example of potent antibody-dependent cellular cytotoxicity (ADCC) mediated by non-neutralizing antibodies, its potential protective role against HIV-1, and evasion strategies used by the virus to circumvent this mechanism of antibody-mediated protection.

## 2. Structural Mechanisms Used by HIV to Evade Antibody-Mediated Protection

HIV-1 can evade neutralizing antibody responses by at least four mechanisms (discussed in [32]). First, highly conserved structures, such as the co-receptor binding domain (CoRBS) of gp120 [32,33,34,35], and most likely the membrane proximal region (MPER) of gp41 [36,37,38], are conformationally masked on pre-fusion trimers of the HIV-1 envelope glycoprotein (Env), becoming unmasked during viral entry. Second, a dense glycan shield shields most of the Env trimer surface and is only penetrated by uncommon bnAbs that use long HCDR3s, extensive somatic hypermutation, or both [39]. Third, marked tolerance for sequence variation in the gp120 hypervariable regions, contributes significantly to neutralization escape (reviewed in [40]). Fourth, the low density of Env trimers on virions disfavors neutralization by inter-spike cross-linking (*cf*. [41,42]). In contrast to neutralization, much less is known about the evasion of protection by antibodies that depend partially or exclusively on Fc-mediated effector function. In the following paragraphs, we summarize the structural basis for an additional example of conformational masking for non-neutralizing antibodies specific for highly conserved epitopes of the gp41 interactive face of gp120, which mediate potent antibody-dependent cellular cytotoxicity (ADCC) against target cells sensitized by entering virions [29,43] or by virion budding [44,45].

### Conformational Changes in Env during Viral Entry and Budding

It is well established the HIV envelope glycoprotein (Env) undergoes a discrete series of conformational changes during viral entry, traversing an energy landscape that leads ultimately to viral and cell membrane fusion (reviewed in [46,47,48]). These conformational changes require the stepwise interaction between Env on the virion surface and CD4 on the target cell surface, forming an Env-CD4 complex that interacts with either CCR5 or CXCR4, enabling viral-cell membrane fusion that is mediated by gp41 rearrangements. Recent structural and biophysical studies are shedding new light on the nature of these conformational changes, their relationships to viral entry, and their role in antibody-mediated protection against HIV-1 [32,33,34,35,49]. Intra-spike fluorescence energy transfer (FRET) studies [35,49] confirm the long-suspected view that Env is conformationally dynamic on the viral membrane [50]. The FRET studies also suggest that the conformational states spontaneously sampled by unliganded Env trimers correspond to states that are stabilized by receptor and co-receptor interactions during viral entry [35,49]. Similar conclusions were reached in deuterium-exchange studies (HDX) using soluble Env trimer mimetics [49,51]. Taken together, these studies provide an increasingly clear picture of the conformational dynamics of Env as it traverses the energy landscape leading to viral and cell membrane fusion. By contrast, much less is known about the conformational changes in Env during virus assembly and budding.

HIV-1 buds from cells largely as “immature” particles requiring cleavage of Pr55^Gag^ via the viral protease (reviewed in [52]). Cleavage is essential for capsid maturation, rendering the virion infectious. Pr55^Gag^ is targeted to the inner cell membrane by the matrix protein (MA) that also interacts with the long cytoplasmic tail of gp41. This interaction affects the conformation of the ectodomain of Env that results in poor fusogenicity of viral spikes until MA is cleaved from Pr55^Gag^ by the viral protease during virus maturation [36,53,54,55,56]. The conformational masking of the gp41 MPER region recognized by bnAbs is an immunologic correlate of this change in Env conformation during virus maturation [36]. The nature of these changes in the Env ectodomain and how they are controlled by interaction of the Env cytoplasmic tail with MA are unknown and constitute a significant area of research that remains largely unplumbed. 

## 3. Identification of Epitope Cluster A of gp120 as a Target for Potent Fc-Mediated Effector Function

Fc-mediated effector function, especially ADCC, against HIV infected cells has been studied since early in the epidemic [57,58,59,60]. A number of studies, both in HIV-1 infected individuals and SIV/SHIV infected rhesus macaques, strongly support a role for this mechanism in the post-infection control of viremia (reviewed in [28]). There is also suggestive evidence that Fc-mediated effector function is involved the prevention of HIV acquisition, which is an open and controversial question (reviewed in [28]). Regardless of this controversy, it has been known for many years that antibodies to both gp120 [59] and gp41 [61] can mediate ADCC against infected cells. Until recently, however, there have been few quantitative studies to define the relationships between epitope specificity and the potency of Fc-mediated effector functions. Our interest in this problem stems from a confluence of studies over the years on epitope imaging Env-mediated membrane fusion [38,62,63], protection in both human [64,65] and NHP [66,67,68,69] vaccine studies, and the role of antibodies in post-infection control of viremia in HIV infected people [70,71,72,73,74,75,76,77,78,79,80,81,82,83,84,85,86,87,88,89]. Protection in our NHP vaccine studies, using a conformationally constrained gp120 [90], correlated directly with antibody specificity for CD4-induced epitopes (CD4i epitopes) [66,67,68] and with ADCC [67,68] measured in the rapid fluorescent ADCC (RFADCC) assay [91]. Protection correlated inversely with the magnitude of antigen-specific T cell responses [67], showing that protection requires not only the correct antibody response but also a “balanced” T cell response. A “balanced” T cell response must provide sufficient help for antibody production by B cells without creating sufficient new CD4+ T cell targets that provide a more fertile environment for viral replication, blunting protection (discussed in [21] and demonstrated experimentally in [92]). Based on these observations, we initiated a series of studies to define the CD4i epitope specificities in our antibody binding and effector assays that correlate with protection in our NHP studies. 

This work led to the identification Epitope Cluster A in gp120 as a potent target of antibodies that mediate ADCC against target cells sensitized with entering virions [43]. Epitope Cluster A is located in the gp41-interactive region of gp120 [43,93] and it is comprised of two subregions defined by canonical mAbs. The first is A32, which recognizes an epitope associated primarily with the C1 and C2 regions [93,94,95,96]. The A32 subregion was shown to be highly immunogenic in HIV infected individuals [45], and RV144 vaccinees [65,97,98], where it is a target of antibodies that mediate ADCC against infected target cells [44,45]. Further, we recently reported that the A32-subregion is a target of vaccine-elicited antibodies in rhesus macaques where protection against repeat, low-dose rectal challenges with SHIV162P3 correlates with binding antibodies to specificity as well as with ADCC [67]. The second is C11, which recognizes an epitope mapping to the 7-stranded β-sandwich of gp120 [95,96,99]. We have solved the X-ray crystal structure of the A32 subregion [93] and developed a docking model for the C11 subregion to provide insight into the potential mechanisms by which antibodies to Epitope Cluster A might protect against HIV-1 and how these responses might be evaded. Although we will discuss the structural basis for Epitope Cluster A in this review, it is not unique as an ADCC target epitope region as ADCC responses are elicited by other epitopes in gp120 [59,81,97,100,101,102,103] as well as gp41 [22,61,103,104]. It is our hope that the information gleaned from the detailed characterization of Epitope Cluster A will be of use in future studies of these other ADCC target epitopes. 

## 4. Structure of the A32 Subregion of Epitope Cluster A

**Figure 1:**
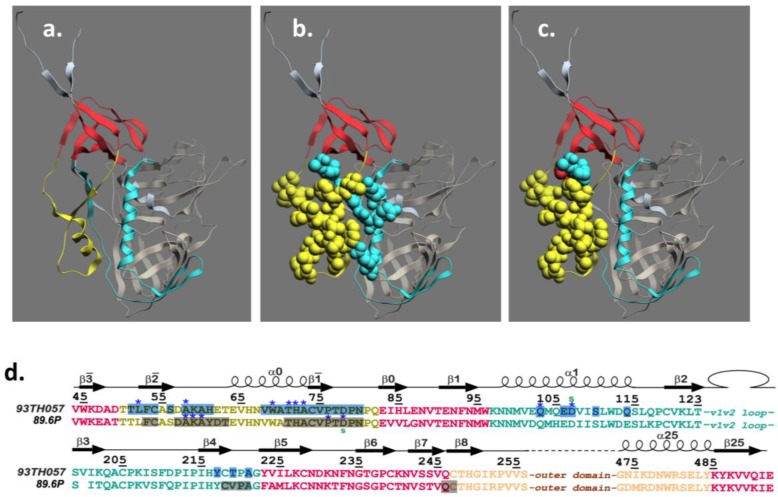
Structure of the A32 sub-region of Epitope Cluster A. Panel (**a**) depicts the mobile layers of gp120 as defined in [95,96] where layer 1 is yellow, layer 2 is cyan, the 7-stranded β-sandwich is red, the gp120 outer domain is bronze and the gp120 inner domain is gray; Panel (**b**) shows epitope contacts for mAb N5-i5 from [93] rendered as cpk structures and colored according to the mobile layers in panel **a**; Panel (**c**) shows epitope contacts for mAb 2.2c from [93] rendered as cpk structures and colored according to the mobile layers in panel **a**; Panel (**d**) shows the relationships between epitope contact residues for mAb N5-i5 binding to gp120_93TH057_ and 2.2c binding to gp120_89.6P_ from [93]. The coloring scheme for mobile layers 1 and 2 as well as the 7-stranded β-sandwich are the same as for panel **a**.

We recently reported the first structures of the A32 subregion of epitope Cluster A using two mAbs, N5-i5 [43,93] and 2.2c [93]. These mAbs differ in ADCC potency where N5-i5 is approximately 75-fold more potent than 2.2c as defined by EC50, the mAb concentration in nM required for half-maximal activity [93]. Both mAbs exhibit the same level of maximal cytotoxicity in our ADCC assay [93]. In addition to defining the A32 epitope subregion at the atomic level, our studies provided the first insight into how the mechanism of mAb binding to the target epitope affects ADCC potency [93]. As shown in Figure 1 N5-i5 contacts gp120 between mobile layer 1 (panels a, b, d) (β$\bar{2}$-, β$\bar{1}$-strands, α0-helix and β$\bar{2}$-α0-, β$\bar{1}$-β0-connecting coils; residues: 51–54, 56, 58–61 and 68–80) and mobile layer 2 (α1-helix, β4-strand and β4-β5-connecting coil; residues: 103, 106–107, 110, 114, 217, 219 and 221) of the C1 and C2 regions. Thus, 82% of the epitope footprint maps to mobile layer 1 [95,96] and 18% maps to mobile layer 2. The paratope of N5-i5 is relatively flat, with a short HCDR3 (10 residues), and electropositive, forming a precise complementary interaction with the electronegative paratope [93]. In addition, N5-i5 is only modestly mutated in comparison with bnAbs that are most often highly mutated [20]. Interestingly, the epitope recognized by 2.2c is overlapping but distinct from the epitope recognized by N5-i5 (Figure 1). Ninety-four percent of the epitope footprint for 2.2c maps to mobile layer 1 using most of the same contacts used by N5-i5 (Figure 1, panels c and d). Unlike N5-i5, 2.2c gp120-bound structures of A32 and two additional A32-like mAbs and the epitope footprints are highly overlapping with the N5-i5 epitope involving both layer 1 and layer 2 contacts (in preparation). Thus, the 2.2c epitope appears to be a structural outlier in the A32 epitope subregion. It is also a functional outlier in that 2.2c is the least potent of our A32-like mAbs, which are all similar to N5-i5 in ADCC potency [43,93]. The unusual structural and functional properties of 2.2c suggested that its distinct mode of binding might account for its modest potency compared with N5-i5 and the other A32-like mAbs. 

**Figure 2:**
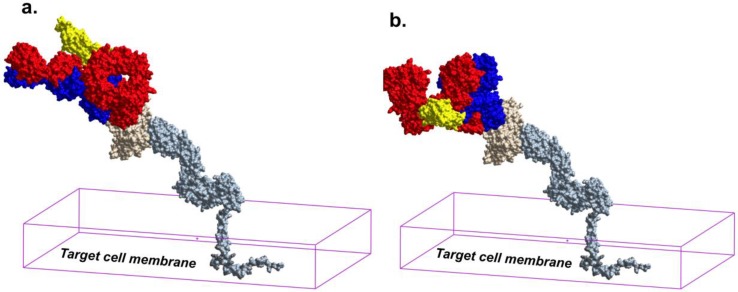
Epitope binding angle determines the orientation of IgG1 toward or away from Fc-receptors on the effector cell membrane. Panel (**a**) shows the predicted orientation of the CH2 domain of N5-i5 IgG1 when it is bound to a gp120-CD4 complex on the target cell membrane facilitating its recognition by an FcγR on the effector cell membrane (from [93]). Four domain cell surface CD4 was generated using PDB:1WIO (ectodomain) and PDB:2KLU (transmembrane and cytoplasmic domain). The N5-i5 complex with gp120 used PDB:4H8W and 2.2c complex with gp120 used PDB:4R4F. The 7S IgG1 structure was from PDB:1GY and the human FcγR3a-human IgG1 Fc complex structure was from PDB:1E4K. Cell surface CD4 is colored as steel, gp120 as beige, IgG1 heavy chain as red, light chain as blue, and FcγR3a is yellow. The parallelogram represents the target cell membrane. The figure was constructed using ICMPro, Molsoft, LLC, La Jolla, CA, USA; Panel (**b**) shows the predicted orientation of the CH2 domain of 2.2c IgG1 when it is bound to a gp120-CD4 complex on the target cell membrane disfavoring its recognition by an FcγR on the effector cell membrane (from [93]).

## 5. Structure of the C11 Subregion of Epitope Cluster A

**Figure 3:**
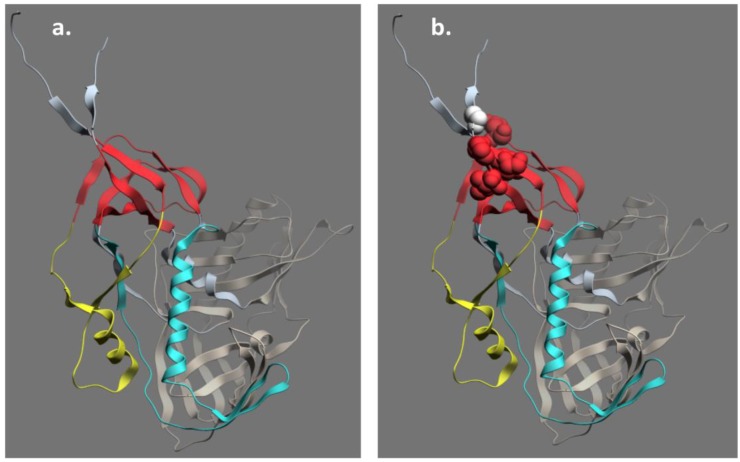
Structure of the C11 sub-region of Epitope Cluster A. Panel (**a**) shows the mobile layers of gp120 as defined in [95,96] where layer 1 is yellow, layer 2 is cyan, and the 7-stranded β-sandwich is red, the gp120 outer domain is bronze and the gp120 inner domain is gray; Panel (**b**) shows residues 45, 88, 491,493, and 495 that are putative contact sites for C11 [96,99]. The putative C11 contact residues map into the 7-stranded β-sandwich and the C-Terminal extension of the gp120.

Much less is known about the structure of the C11 subregion of Epitope Cluster A although it is a very potent ADCC target in multiple assay formats [43,44]. Early studies suggested that the C11 epitope maps to the C1 and C5 regions of gp120 [99]. Further studies confirmed this result placing the C11 epitope in the 7-stranded β-sandwich of gp120 and C-Terminal extension of gp120 (Figure 3) ([95,96] and submitted). We have solved the structure of unliganded C11 and developed a docking model that places the C11 epitope on the same face of gp120 as the A32 epitope subregion but without overlap (submitted). While this model is being tested in additional crystallization trials, it is consistent with our aggregate data and suggests an additional example of conformational epitope masking by which HIV-1 evades a potentially protective antibody response. As discussed above, the CoRBS and MPER are examples of conformationally masked epitopes recognized by neutralizing antibodies, which can also mediate ADCC in many cases [43,105,106,107]. 

## 6. Epitope Cluster A Is Conformationally Masked on Virions and Partially Formed in Unliganded Soluble Env Trimer Analogs

Our recent studies using fluorescence correlation spectroscopy and confocal/superresolution microscopy show that Epitope Cluster A is buried on virion-associated unliganded Env trimers [108] and becomes exposed during viral entry where it remains exposed long enough to sensitize target cells for ADCC [29,43,63]. Surprisingly, epitope Cluster A can also be exposed during virus budding provided sufficient cell surface CD4 is present to trigger its exposure, rendering even infected cells susceptible to killing by A32-like and C11-like antibodies [44,45]. In unliganded soluble SOSIP trimer analogs Epitope Cluster A maps precisely to the regions of gp120 that dock into gp41 during biosynthesis to form stable Env trimers on the viral membrane (Figure 3), which is also consistent with mutagenesis [96,109,110,111,112,113,114] and structural studies of gp120 monomers [95,96] and cryo-electron microscopy of native Env trimers on virions (reviewed in [115]). By analogy with the highly conserved receptor binding domains of gp120, gp41 can be viewed as a transmembrane “receptor” for gp120 where their non-covalent association occurs during biosynthesis. Of necessity, both the “ligand” elements of gp120 and the “receptor” elements of gp41 are highly conserved, which is likely why they are masked in native Env trimers and unmasked only by the conformational changes that occur consequent to receptor binding. Epitope Cluster A exposure is a consequence of these conformational changes.

**Figure 4:**
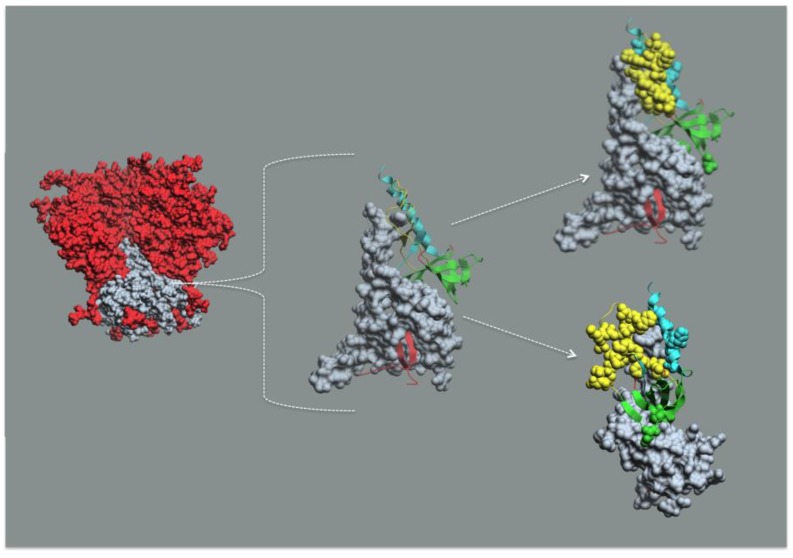
Epitope Cluster A maps into the gp41 docking site for gp120 in Env trimer mimetic structures. The leftmost structure is the soluble SOSIP Env timer mimetic from PDB:4TVP [95] where gp120 is red and gp41 is gray. The middle figure is a gp41 monomer from PDB:4TVP in gray. The gp41 interactive face comprised of elements from mobile layer 1 (yellow), mobile layer 2 (cyan), the 7-stranded β-sandwich (green), and the N- and C-Terminal extensions (red) of monomeric gp120 shown as ribbon diagrams. The upper rightmost figure is the same as the middle figure except with the N5-i5 and C11 contact residues rendered as cpk structures. The lower rightmost figure is the same as the upper rightmost figure rotated approximately 90°. Note that the N5-i5 contact residues are in mobile layers 1 and 2 (yellow and cyan), whereas the C11 contact residues are in the 7-stranded β-sandwich (green). The viral membrane would be at the bottom of each structure.

In this context, it is useful to consider the structural basis of the gp41 “receptor”, the gp120 “ligand” interaction, and their relationships to Epitope Cluster A. As shown in Figure 4, gp120 is non-covalently bound to gp41 where the sheets of its N- and C-Terminal extensions insert through a hydrophobic four-helix collar formed by the α6, α7, α8, and α9 helices of gp41 facing toward the viral membrane. gp120 “docks” into the gp41 “receptor” by interactions of the 7-stranded β-sandwich and N- and C-Terminal extensions of gp120 with the four-helix collar of gp41. The docking of gp120 into gp41 also involves contacts between the loop connecting the β$\bar{2}$-, β$\bar{1}$-strands of mobile layer 1 of gp120 with α-7 helix of prefusion gp41 (or HR1 in post-fusion gp41) that protrudes above the four-helix collar. Thus, the A32 epitope subregion binds the α7 helix of gp41 and the C11 epitope subregion binds the four-helix collar of gp41. These interactions place Epitope Cluster A deep within the unliganded SOSIP Env trimer analog. It is possible that the I559P mutation results in an altered gp41 conformation in the unliganded SOSIP trimer [116] and these details might need to be revised as other structures become available. Nevertheless, based on the collective data from mutagenesis, X-ray crystallography, and cryo-electron microscopy discussed above, it is safe that Epitope Cluster A is buried within the trimer axis. The thermodynamics for Epitope Cluster A masking and unmasking are not known but it is likely that the biosynthetic energy required to form a stable docking of gp120 into gp41 in the trimer context comprises a significant fraction of the energy required for viral-cell membrane fusion. There is an additional element of conformational masking of Epitope Cluster A. The α0 helix is disordered in the Env trimer disrupting the local structure of the A32 epitope subregion as shown in [93]. Taken together, these studies show that conformational masking restricts access to this highly conserved target of non-neutralizing antibodies that mediate potent ADCC. The question becomes that of whether receptor-dependent conformational unmasking of these epitopes provides a sufficient window of opportunity anti-Epitope Cluster A antibodies to impinge HIV-1 infection.

## 7. Receptor Dependent Conformational Unmasking of Epitope Cluster A

Elsewhere, we have discussed in detail the relationships between antibody responses to Epitope Cluster A and protection against HIV-1 acquisition and in post-infection control [28,29]. Here, we will focus on the points during acquisition and post-infection control where Epitope Cluster A becomes unmasked and susceptible to immune attack by antibodies via Fc-mediated effector function. There are two windows of opportunity for antibodies to Epitope Cluster A to reduce HIV-1 infection, during viral entry and viral budding. As pointed out above, we showed recently that Epitope Cluster A is essentially absent from the surfaces of virions [108] but is exposed shortly after binding to cell surface CD4 [63,93] and remains exposed for at least two hours [63], during which the target cell is susceptible to killing by effector cells and anti-Epitope Cluster A antibodies [43]. Our recent study suggests that after virion binding, Epitope Cluster A becomes highly exposed distal to the virological synapse rendering the target cells highly sensitive to Fc-mediated effector function [63]. Thus, the window of opportunity for killing nascently infected cells by anti-Epitope Cluster A antibodies is open for at least two h and probably longer. This time may seem too short to be of significance in blocking HIV-1 infection; however, it is important to note that passive immunization studies suggest that the effective window of opportunity for neutralizing antibodies to block acquisition is no longer than 24 h [4,117]. In this context, passive transfer of non-neutralizing antibodies has not blocked high-dose challenges with SHIV [14,22]. However, it has been reported that A32 as well as a non-neutralizing antibody specific for the gp41 disulfide loop can reduce the number of transmitted SHIV variants in a passive immunization study [118]. The conformational unmasking of Epitope Cluster A occurs as a consequence of stored energy being released consequent to viral entry. There is a largely unappreciated role of cell surface CD4 in the unmasking Epitope Cluster A. We have been unable to expose Epitope Cluster A on free virions using soluble CD4 although this interaction clearly exposes the CoRBS [108]. By contrast, Epitope Cluster A becomes exposed within 5 min after binding to cell surface CD4 [63]. Thus, cell surface CD4 makes an energetic contribution to Env trimer rearrangement that is not recapitulated by soluble CD4. Cell surface CD4 exists as monomers and dimers that can interconvert by redox reactions where a reduced monomer appears to be the primary form used for HIV-1 entry and an oxidized dimer is the primary form for Class II MHC mediated antigen presentation [119,120,121,122,123]. Collectively, these studies point toward an underappreciated role for cell surface CD4 in Epitope Cluster A exposure and target cell sensitization in addition to viral entry that deserves additional study. 

On the other end of the viral replicative cycle, Epitope Cluster A is also exposed during viral budding from infected cells, rendering them sensitive to killing by ADCC [44,45]. In this instance, Epitope Cluster A is unmasked by the *cis* interaction of Env trimers with residual CD4 on the infected cell surface [44]. The Env-CD4 interaction is modulated by the HIV-1 accessory proteins Nef and Vpu, which are known to decrease cell-surface levels of CD4 [124,125]. In addition to its role in CD4 degradation, Vpu also antagonizes a restriction factor, Tetherin/BST-2, which normally inhibits retroviral release [126,127]. Viruses lacking Vpu remain trapped at the cell surface resulting in an accumulation of exposed Env [44,128,129,130]. Therefore, Nef and Vpu can indirectly modulate Env-CD4 interaction at the surface of infected cells through CD4 and BST-2 downregulation [44,128]. Cells infected with viruses defective for both Nef and Vpu present enhanced levels of CD4 and Env at the cell-surface, resulting in the exposure of Epitope Cluster A rendering the cells sensitive to killing by antibodies to this region [44,128]. However, the vast majority of circulating HIV-1 strains worldwide express functional Nef and Vpu proteins, likely limiting the exposure of CD4i Env epitopes at the surface of infected cells and thus preventing ADCC responses. Therefore, targeting Vpu and Nef ability to down-regulate CD4 and BST-2 or strategies aimed at modifying Env conformation to expose CD4i epitopes could potentially render HIV-1-infected cells susceptible to ADCC and thus have therapeutic utility. In this sense, agents promoting the CD4-bound Env conformation should expose CD4i epitopes that are readily recognized by ADCC-mediating Abs present in sera and cervicovaginal lavages (CVLs) from vaccinated and infected individuals [44,128,131,132,133]. Importantly, modulating Env conformation at the surface of HIV-1-infected cells has become feasible as a result of the availability of small CD4-mimetic compounds. The prototypes of such compounds, NBD-556 and NBD-557, were discovered in a screen for inhibitors of gp120-CD4 interaction [134]. These small-molecule ~337-dalton compounds and recent derivatives (DMJ-I-228) bind in the Phe 43 cavity [135,136,137], a highly conserved ~150-Å^3^ pocket in the gp120 glycoprotein located at the interface of the inner domain, outer domain, the bridging sheet and the CD4 receptor [138]. CD4-mimetics block gp120-CD4 interaction and induce thermodynamic changes in gp120 similar to those observed upon soluble CD4 (sCD4) binding [139]. Accordingly, these small molecules as well as sCD4 can promote the transition of Env to the CD4-bound conformation, thus sensitizing HIV-1 particles to neutralization by otherwise non-neutralizing CD4i Abs [140,141]. 

Additional strategies using scaffolded miniproteins targeting critical gp120 elements required for CD4 interaction allowed the identification of CD4-mimetics with nanomolar affinity for gp120 [142]. One of these variants, M48U1, displayed remarkably potent neutralization of three HIV-1 isolates [143]; its crystal structure in complex with HIV-1 gp120 was recently solved, showing that M48U1 engages the Phe 43 cavity in a manner similar to that of cell surface CD4 [144]. Thus, CD4-mimetics might induce gp120 to adopt the CD4-bound conformation, expose CD4i epitopes at the surface of infected cells and thus sensitize them to ADCC-mediated killing. In a recent study we were able to sensitize HIV-1-infected cell to ADCC killing mediated by autologous and heterologous sera [145]. . However, whether this was mediated by Epitope Cluster A antibodies present in the sera remains to be determined. In this context, it will be important to determine which, if any, of these small molecules expose Epitope Cluster A in addition to epitopes recognized by neutralizing antibodies. 

## 8. Conclusions

In addition to protection mediated by broadly neutralizing antibodies, the studies cited above strongly suggest that the exposure of Epitope Cluster A represents an additional window of opportunity for antibodies to interdict HIV-1. This protection requires Fc-mediated effector function because anti-Epitope Cluster A antibodies are uniformly non-neutralizing. It is useful to frame the discussion by the length of time that the window of opportunity is open for protection by different mechanisms of antibody-mediated protection against HIV-1. Most of the potent broadly neutralizing antibodies recognize epitopes constitutively exposed on infectious virions and neutralize by stabilizing the conformation of unliganded Env [49,108,146,147,148]. Since the window of opportunity for these antibodies is determined solely by epitope exposure on infectious virions, it is relatively wide, which is reflected in ability of bnAbs to protect against high-dose SHIV challenges *in vivo* [7,9,10], although this window appears to be less than 24 h ([4,117] and reviewed in [28,29]). By contrast, the window of opportunity for antibodies to Epitope Cluster A is shorter, most likely on the scale of a few hours following cell surface CD4 binding [43,63]. This is consistent with the inability of A32 to protect against a high-dose SHIV challenge, although it does reduce the number of transmitted variants as reported in [118]. The possibility remains that antibodies to Epitope Cluster A might be sufficient to protect against low-dose SHIV challenges. This is suggested in our recent repeat low-dose SHIV challenge study where protection against acquisition correlated with both ADCC titers and binding antibody titers to the A32 epitope subregion [67]. In contrast to bnAbs, the ease with which anti-Epitope Cluster A antibodies are elicited by vaccination [65,66,67] supports continued exploration of this region of gp120 as a target for protection against HIV-1 acquisition. Finally, the high frequency of responses to Epitope Cluster A in HIV-1 infected people [43,45], coupled with the possibility of using small molecule drugs and CD4-miniprotein mimetics to expose this region on infected cells [145] offers a unique opportunity to increase post-infection control of viremia and possibly even to reduce or clear the latent reservoir. 
